# Network Pharmacology and Experimental Verification Strategies to Illustrate the Mechanism of Jian-Pi-Yi-Shen Formula in Suppressing Epithelial–Mesenchymal Transition

**DOI:** 10.3389/fphar.2022.873023

**Published:** 2022-05-17

**Authors:** Yuan Zhao, Xiangbin Li, Fochang Wang, Shiying Huang, Hanqian Du, Shunmin Li, Jianping Chen

**Affiliations:** ^1^ Shenzhen Key Laboratory of Hospital Chinese Medicine Preparation, Shenzhen Traditional Chinese Medicine Hospital, The Fourth Clinical Medical College of Guangzhou University of Chinese Medicine, Shenzhen, China; ^2^ Shenzhen Hospital of Beijing University of Chinese Medicine (Longgang), Shenzhen, China; ^3^ Institute of Chinese Materia Medica, China Academy of Chinese Medical Sciences, Beijing, China

**Keywords:** Jian-Pi-Yi-Shen formula, renal fibrosis, epithelial–mesenchymal transition, network pharmacology, Wnt3a/β-catenin signaling pathway

## Abstract

Jian-Pi-Yi-Shen formula (JPYSF), a traditional Chinese medicine, has been recommended to treat renal fibrosis for decades. Previous studies had shown that JPYSF could inhibit epithelial–mesenchymal transition (EMT), an important regulatory role in renal fibrosis. However, the mechanism of JPYSF action is largely unknown. In this study, network pharmacology and experimental verification were combined to elucidate and identify the potential mechanism of JPYSF against renal fibrosis by suppressing EMT at molecular and pathway levels. Network pharmacology was first performed to explore the mechanism of JPYSF against renal fibrosis targeting EMT, and then a 5/6 nephrectomy (5/6 Nx)-induced rat model of renal fibrosis was selected to verify the predictive results by Masson’s trichrome stains and western blot analysis. Two hundred and thirty-two compounds in JPYSF were selected for the network approach analysis, which identified 137 candidate targets of JPYSF and 4,796 known therapeutic targets of EMT. The results of the Gene Ontology (GO) function enrichment analysis included 2098, 88, and 133 GO terms for biological processes (BPs), molecular functions (MFs), and cell component entries, respectively. The top 10 enrichment items of BP annotations included a response to a steroid hormone, a metal ion, oxygen levels, and so on. Cellular composition (CC) is mainly enriched in membrane raft, membrane microdomain, membrane region, etc. The MF of JPYSF analysis on EMT was predominately involved in proximal promoter sequence-specific DNA binding, protein heterodimerization activity, RNA polymerase II proximal promoter sequence-specific DNA binding, and so on. The involvement signaling pathway of JPYSF in the treatment of renal fibrosis targeting EMT was associated with anti-fibrosis, anti-inflammation, podocyte protection, and metabolism regulation. Furthermore, the *in vivo* experiments confirmed that JPYSF effectively ameliorated interstitial fibrosis and inhibited the overexpression of α-SMA, Wnt3a, and β-catenin, and increased the expression of E-cadherin by wnt3a/β-catenin signaling pathway in 5/6 Nx-induced renal fibrosis rats. Using an integrative network pharmacology-based approach and experimental verification, the study showed that JPYSF had therapeutic effects on EMT by regulating multi-pathway, among which one proven pathway was the Wnt3a/β-catenin signaling pathway. These findings provide insights into the renoprotective effects of JPYSF against EMT, which could suggest directions for further research of JPYSF in attenuating renal fibrosis by suppressing EMT.

## Introduction

Renal fibrosis can lead to the progressive loss of renal function, with the characteristic of the proliferation of renal intrinsic cells, activation of renal interstitial fibroblasts, and deposition of extracellular matrix, which is the most common cause of kidney failure (Zhao et al., 2021). A large part of interstitial myofibroblasts, the main effector cells of renal fibrosis, are derived from tubular epithelial cells of the affected kidneys via epithelial–mesenchymal transition (EMT) (Hu et al., 2021). EMT, one of the main mechanisms of renal fibrosis, had been found to contribute to ongoing fibrosis in kidney disease through a variety of signal pathways, which ultimately led to renal function impairment, and then developed into chronic renal disease and end-stage renal disease. Despite extensive studies, the current treatment strategies of western medicine can hardly reverse the progression of renal fibrosis, underscoring the need to develop an alternative therapeutic approach to reverse or stop the progression. Traditional Chinese medicine (TCM), with the feature of multi-component, multi-target, and multi-channel, can enhance body functions and reduce drug toxicity through the synergistic actions of their main active ingredients, which could be a rich source for drug discovery. The experimental models and clinical studies have proved that TCM and its extracts have made great progress in the prevention or even treatment of renal fibrosis, and effectively inhibited the development of EMT in recent years (Xu et al., 2021).

Jian-Pi-Yi-Shen Formula (JPYSF) is derived from the addition and subtraction of “Yupingfeng San” and “Sijing Pills” (recorded in Shengji Zonglu). JPYSF has been prescribed for patients with chronic kidney disease in clinics, which consists of *Astragalus mongholicus* Bunge (*A. mongholicus*), *Dioscorea oppositifolia* L. (*D. oppositifolia*.), *Salvia miltiorrhiza* Bge. (*S. miltiorrhiza*), *Atractylodes macrocephala* Koidz. (*A. macrocephala*), *Cistanche deserticola* Ma (*C. deserticola*), *Amomum kravanh* Pierre ex Gagnep. (*A. kravanh*), *Rheum palmatum* L. (*R. palmatum*), and *Glycyrrhiza uralensis* Fisch. (*G. uralensis*). Adopting the two-complement five-way method, the two complement mainly consists of “tonifying the kidney” and “tonifying the spleen.” *A. mongholicus*, *A. macrocephala*., *D. oppositifolia*., and *C. deserticola* were used as spleen and kidney supplements (Zhong et al., 2016). *A. mongholicus* was used for nourishing the qi of the five viscera, and it was an important medicine for nourishing the qi of the spleen and kidney. “Five-way” means to make sure that feces, urine, sweat, breath, and blood are unobstructed. *R. palmatum*, *A. macrocephala*, and *C. deserticola* were used to pass bowel movements, having the functions of loosening the bowel to relieve constipation (Zhong et al., 2016). *A. mongholicus* and *A. macrocephala* were used for urination, having a diuretic effect (Fu et al., 2014). *A. kravanh*, which has the effect of warming middle energizer, dissipating hygrosis and regulating *qi*, and promoting sweat and breathing well, was used to drain sweat and breath. *S. miltiorrhiza* and *R. palmatum* were used to promote blood circulation, having the effect of promoting blood circulation and removing blood stasis (Mei et al., 2019; Pei et al., 2020). *G. uralensis* was used for invigorating the spleen and harmonizing herbs. Taken together, JPYSF, the combined use of all the above-mentioned herbs, has the effects of invigorating the spleen and kidney, promoting blood circulation, and removing turbidity. Previous pharmaceutical studies have revealed that JPYSF had effectiveness in the treatment of chronic kidney disease and renal fibrosis (Liu et al., 2018; Lu et al., 2018; Chen et al., 2019). However, the composition of JPYSF is complex and its mechanism of action is not clear enough, which limits its wide clinical application.

Network pharmacology is a theory based on systems biology and has been commonly used in the modern research of TCM in recent years. It emphasizes the multichannel regulation of signaling pathways, which is in accord with the characteristics of multi-components and multi-targets of Chinese medicine. Therefore, network pharmacology has become a new effective approach in TCM research at the molecular level (Zhang et al., 2021).

In this study, the integrated strategy of network pharmacology and verification *in vivo*, we first explored the main active ingredients, targets, and signal pathways of JPYSF in the treatment of EMT, and then the animal experiment was carried out to verify the effect of JPYSF against renal fibrosis by inhibiting EMT. The research may lay a good theoretical foundation for further study on developing new drugs for renal fibrosis.

## Methods

### Network Pharmacology Analysis

#### Determination of the Active Components of Jian-Pi-Yi-Shen Formula

All chemical ingredients from the eight herbal medicines of JPYSF were collected from an online database, including a traditional Chinese medicine system pharmacology database and an analysis platform (TCMSP, https://tcmspw.com/tcmsp.php), Integrative Pharmacology-based Research Platform of Traditional Chinese Medicine (TCMIP) v2.0 (Xu et al., 2019; Su et al., 2021), and previous pieces of literature (Wang F et al., 2020). All chemical ingredients were employed to evaluate the degree of drug absorption based on the criteria of oral bioavailability (OB ≥ 30%) and drug-like (DL ≥ 0.18) (mean value for all molecules within the DrugBank database).

### Target Screening of Active Components of Jian-Pi-Yi-Shen Formula and Epithelial–Mesenchymal Transition

The target screening of components was predicted using TCMIP v2.0, TCMSP, the Swiss Target Prediction (http://www.swisstargetprediction.ch/) databases, and previous pieces of literature. The candidate’s therapeutic genes related to EMT were acquired from the OMIM (https://www.omim.org/) (Amberger et al., 2019), GeneCards (https://www.genecards.org/), and Drugbank (https://go.drugbank.com/) using “epithelial–mesenchymal transition” as the keyword. Then, all targets were converted into gene names by the UniProt database (https://www.uniprot.org/).

#### Protein–Protein Interaction Network Construction and Analysis

The interaction networks of the common targets of JPYSF and EMT were constructed by the STRING platform (https://www.string-db.org/). To identify the potential hub nodes of JPYSF in the treatment of EMT, the minimum required interaction score was set as the highest confidence 0.9. “hide disconnected nodes in the network” was ticked in network display options. String_interactions.tsv was then exported. Network visualization and analysis were performed using Cytoscape 3.7.1 software.

### Gene Ontology and KEGG Pathway Enrichment Analysis

The coexistent targets of JPYSF and EMT were conducted on VENNY 2.1 website (https://bioinfogp.cnb.csic.es/tools/venny/index.html), and the Venn diagram was then prepared. The coexistent targets were analyzed using R (https://www.r-project.org/) software for the GO and KEGG enrichment analysis. The threshold was set to *p* < 0.05. The results were visually displayed in the form of bubble charts and histograms. The biological processes (BPs), molecular functions (MFs), and cellular compositions (CCs) were included in the GO enrichment analysis.

#### Network Construction of “Chemicals-Shared Target Genes-Signal Pathway”

The network of CTS was constructed with Cytoscape 3.7.1 software. The nodes in the network diagram are targets, chemicals, or signal pathways. The edge means that there is an interactive relationship between a certain target and a certain signal pathway, a component and a certain target, or a certain target and a certain target.

### Experimental Verification

#### Samples and Sample Preparation

All the raw herbs were obtained from Shenzhen Huahui Pharmaceutical Co., Ltd. (Shenzhen, China) and were authenticated by Shangbin Zhang. The extraction of JPYSF was conducted as previously described (Chen et al., 2019). In brief, *A. mongholicus*, *A. macrocephala*, *D. oppositifolia*, *C. deserticola*, *A. kravanh*, *S. miltiorrhiza*, *R. palmatum*, and *G. uralensis* were mixed in the ratio of 30:10:30:10:10:15:10:6. The detailed methods were as described previously (Wang YN et al., 2020). The extract was freeze-dried and stored at −80°C. The powder was re-dissolved in ddH_2_O as a JPYSF sample. The yield of the extract was 30.8%. The extract being used here was chemically analyzed by HPLC-MS, as indicated in Supplementary Figure S1 according to previously established standards (Wang F et al., 2020), which guaranteed the repeatability of biological results.

### Animals

All animal experiments were in accord with the ethics committee of Guangzhou University of Chinese Medicine and the National Institutes of Health Guideline for the care and use of laboratory animals. Forty male Sprague–Dawley (SD) rats weighing between 180 and 220 g were supplied by Guangdong Medical Laboratory Animal Center (Foshan, China). All the rats were housed under controlled conditions (12-h light/12-h dark cycle) in a specific pathogen-free animal facility with free access to rodent food and drinking water. Either 5/6 nephrectomy (5/6 Nx) or sham operation (sham) was performed on SD rats. The 5/6 Nx operation was conducted in a two-step surgery as described previously (Chen et al., 2019). In brief, upper and lower two-thirds of the left kidneys of 30 rats were ablated, and two weeks later, the right kidneys of the animals were removed under anesthesia with sodium pentobarbital (50 mg/kg, intraperitoneal injection). Laparotomy was performed on rats in the sham-operated group, manipulation of the renal pedicles but without destructing the renal tissue, and then replaced intact. Twelve weeks after the second surgery, all rats were randomly classified into four groups for 6 weeks as follows: the sham group that was given a gavage of distilled water, the model group (5/6 Nx) that was given a gavage of distilled water, and the JPYSF group (2.73, 10.89 g/kg) (Liu et al., 2018; Zheng et al., 2020). All rats were anesthetized with sodium pentobarbital by intraperitoneal injection (50 mg/kg). The kidneys were removed and preserved for further analysis.

### Masson’s Trichrome Stains

The kidneys of the rats were fixed with 10% neutral formaldehyde and then paraffin embedding and sectioning were carried out. Pathological changes in the kidney were evaluated by Masson’s trichrome stains. The experimental process and quantitative methods were performed as previously described (Lu et al., 2018).

### Western Blot Analysis

The cortex tissues of the kidneys were lysed with RIPA lysis buffer. Equal amounts of lysates were loaded and electrophoresed through 10% SDS-polyacrylamide gels, and then transferred to the PVDF membrane. Following blocking in 5% non-fat milk for 2 h at room temperature, the membranes were incubated with various primary antibodies at 4°C overnight. The primary antibodies included α-SMA (1:1,000, Cell Signaling Technology, Beverly, MA, United States), E-cadherin (1:1,000, Cell Signaling Technology, Beverly, MA, United States), Wnt3a (1:1,000, Abcam, Cambridge, MA, United States), β-catenin (1:1,000, Abcam, Cambridge, MA, United States), and GAPDH (1:5,000, Abcam, Cambridge, MA, United States). Then, the membranes were incubated in HRP-conjugated secondary antibodies for 2 h at room temperature. HRP activity was visualized using Tanon-6100C (Guangzhou Yuwei Biotechnology Instrument Co., Ltd., Guangzhou, China).

### Statistical Analysis

Data were expressed as mean ± standard deviation. One-way analysis of variance (ANOVA) was used to determine the level of statistical significance followed by Tukey’s multiple comparison tests. There was a significant difference if the ‘*p*’ value was less than 0.05.

## Results

### Network Pharmacology to Illustrate the Mechanism of Jian-Pi-Yi-Shen Formula in Suppressing Epithelial–Mesenchymal Transition

#### Compounds in Jian-Pi-Yi-Shen Formula

There were 232 compounds in JPYSF that were being screened by the conditions of OB value ≥ 30% and DL value ≥ 0.18. Among them, 6 compounds were in *A. macrocephala*, 16 compounds were in *R. palmatum*, 67 compounds were in *S. miltiorrhiza*, 9 compounds were in *A. kravanh*, 89 compounds were in *G. uralensis*, 14 compounds were in *A. mongholicus*, 4 compounds were in *C. deserticola*, and 16 compounds were in *D. oppositifolia*. Luteolin was the common compound derived from both *S. miltiorrhiza* and *A. kravanh*; Jaranol was the common ingredient of *A. kravanh*, *G. uralensis*, and *A. mongholicus*. Mairin and calycosin co-existed in *A. mongholicus* and *G. uralensis*. Quercetin existed in *G. uralensis*, *A. mongholicus*, *A. kravanh*, and *C. deserticola*. (3S,8S,9S,10R,13R,14S,17R)-10,13-Dimethyl-17-[(2R,5S)-5-propan-2-yloctan-2-yl]-2,3,4,7,8,9,11,12,14,15,16,17-dodecahydro-1H-cyclopenta[a]phenanthren-3-ol was present in *A. mongholicus* and *A. macrocephala*. Isorhamnetin, formononetin, and kaempferol were present in *A. mongholicus* and *G. uralensis*. β-sitosterol was present in both *C. deserticola* and *R. palmatum*. Sucrose was present in *A. mongholicus* and *D. oppositifolia*. The aforesaid compounds in JPYSF can be found in Supplementary Table S1.

### Target Collection of Epithelial–Mesenchymal Transition and Active Ingredients of Jian-Pi-Yi-Shen Formula

**FIGURE 1:**
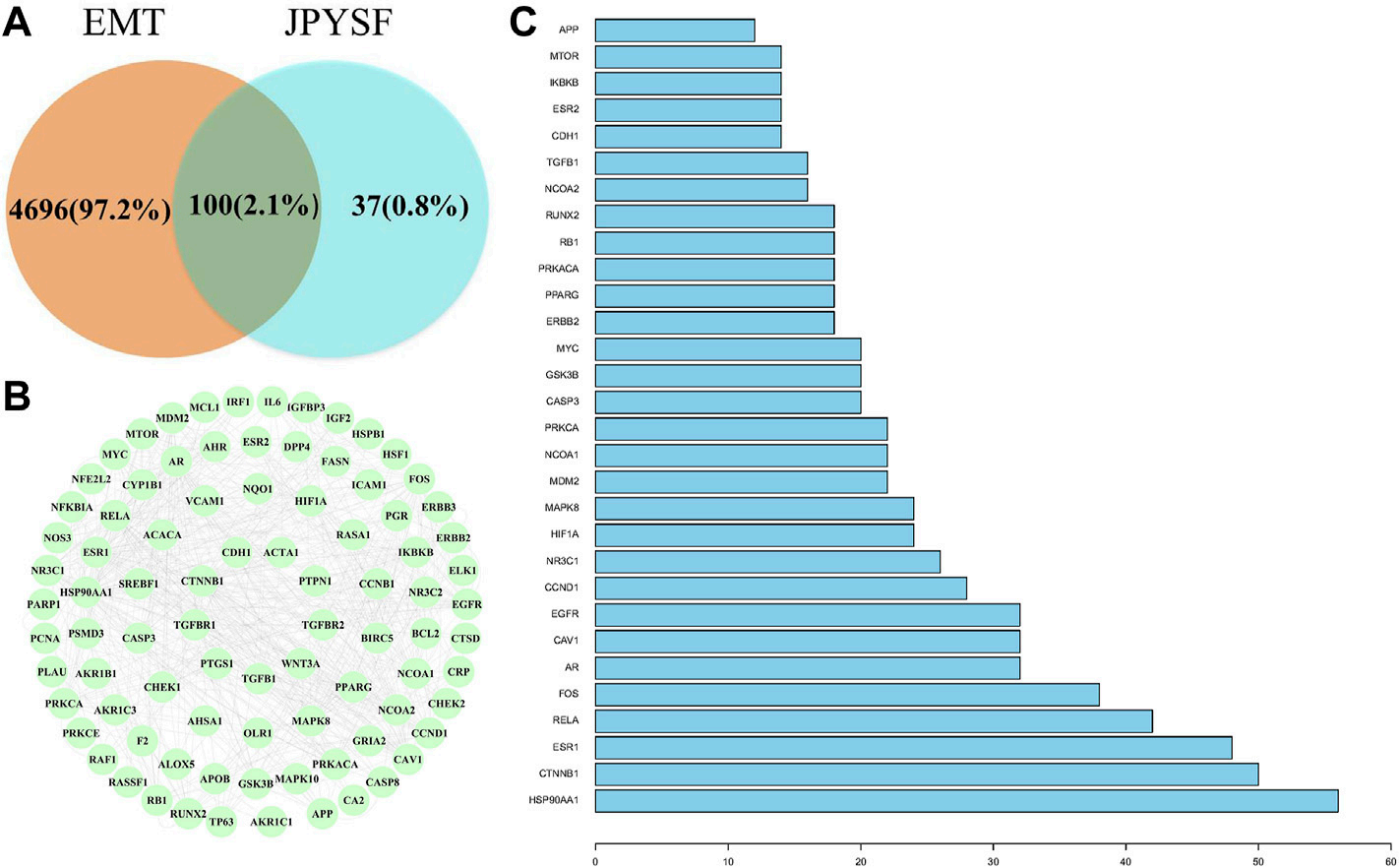
The interaction of shared targets between Jian-Pi-Yi-Shen formula (JPYSF) active ingredients and epithelial–mesenchymal transition (EMT). **(A)**. The Venn diagram; **(B)** 87 connected nodes in the network; and **(C)** The top 30 targets in action frequency.

Fifty-nine EMT-related targets were identified in the DrugBank database, 4,770 targets in the GeneCards database, and 7 targets in the OMIM database. There were 4796 EMT-related targets after removing the duplicate values (Supplementary Table S2). Excluding duplicate targets, a total of 137 candidate targets were queried in JPYSF (Supplementary Table S3). As shown in Figure 1A, there were 100 common targets in both EMT and JPYSF. Among them, 87 targets interacted with others (Figure 1B). HSP90AA1 was the most frequent target, followed by CTNNB1, ESR1, RELA, and FOS. The top 30 targets in action frequency are shown in Figure 1C.

### Gene Ontology Function and KEGG Signal Pathway Enrichment Analysis of Common Targets

**FIGURE 2:**
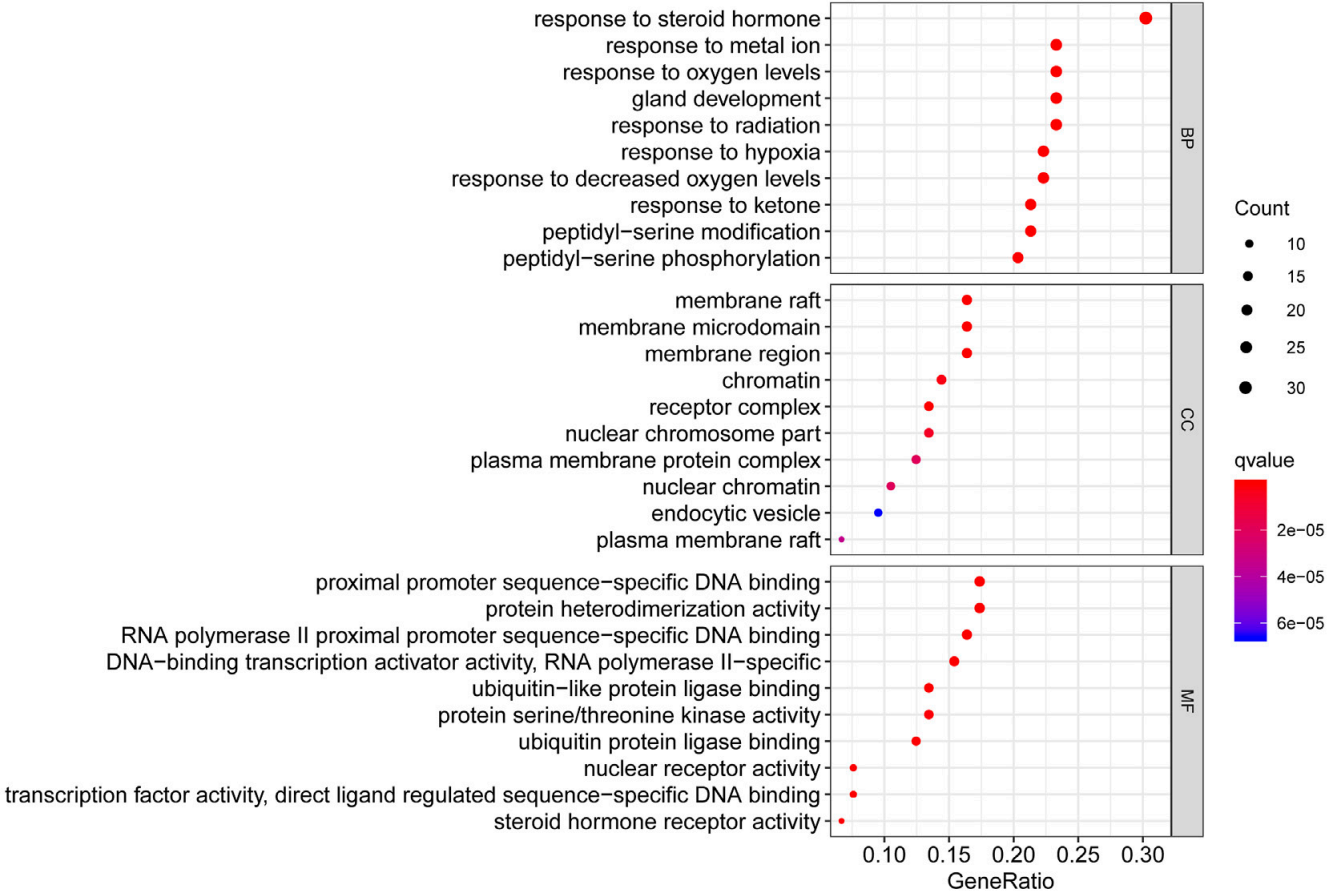
The top 10 pathways for the GO enrichment analysis based on the common targets of the Jian-Pi-Yi-Shen formula (JPYSF) and epithelial–mesenchymal transition (EMT).

**FIGURE 3:**
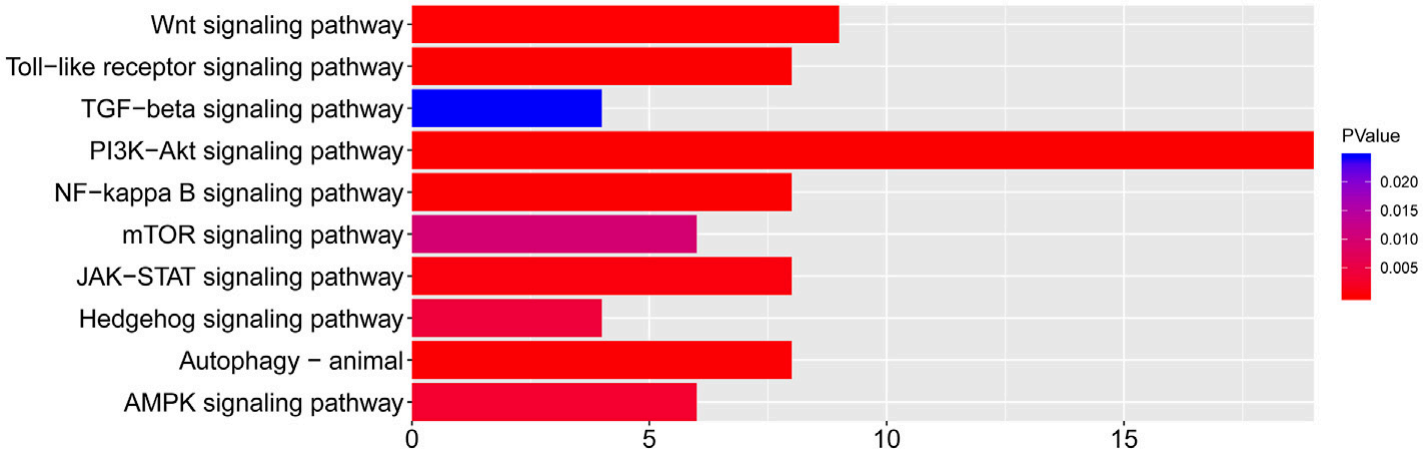
The top 10 pathways for the KEGG pathway are enriched from the common targets between JPYSF and epithelial–mesenchymal transition (EMT).

To better summarize the specific functions of JPYSF in EMT, 100 common targets were imported into R software for GO function annotation and KEGG pathway enrichment. The results showed that a total of 2098 GO terms for BP, 88 GO terms for CC, and 133 GO terms for MF with statistical significance (*p*＜0.05) were obtained (Supplementary Table S4). As shown in Figure 2, the top 10 enrichment items of BP annotations included response to a steroid hormone, response to a metal ion, response to oxygen levels, and so on. CC was mainly enriched in the membrane raft, membrane microdomain, membrane region, etc. The MF of JPYSF analysis on EMT was predominately involved in proximal promoter sequence-specific DNA binding, protein heterodimerization activity, RNA polymerase II proximal promoter sequence-specific DNA binding, etc. Genes that were classified in the pathway analysis were heavily involved in the PI3K-Akt signaling pathway, NF-kappa B signaling pathway, Toll-like receptor signaling pathway, Wnt signaling pathway, JAK-STAT signaling pathway, AMPK signaling pathway, Autophagy–animal, Hedgehog signaling pathway, mTOR signaling pathway, and TGF-beta signaling pathway (Figure 3). Genes enriched in signaling pathways are demonstrated in Supplementary Table S5.

### Network Construction of CTS and Analysis

**FIGURE 4:**
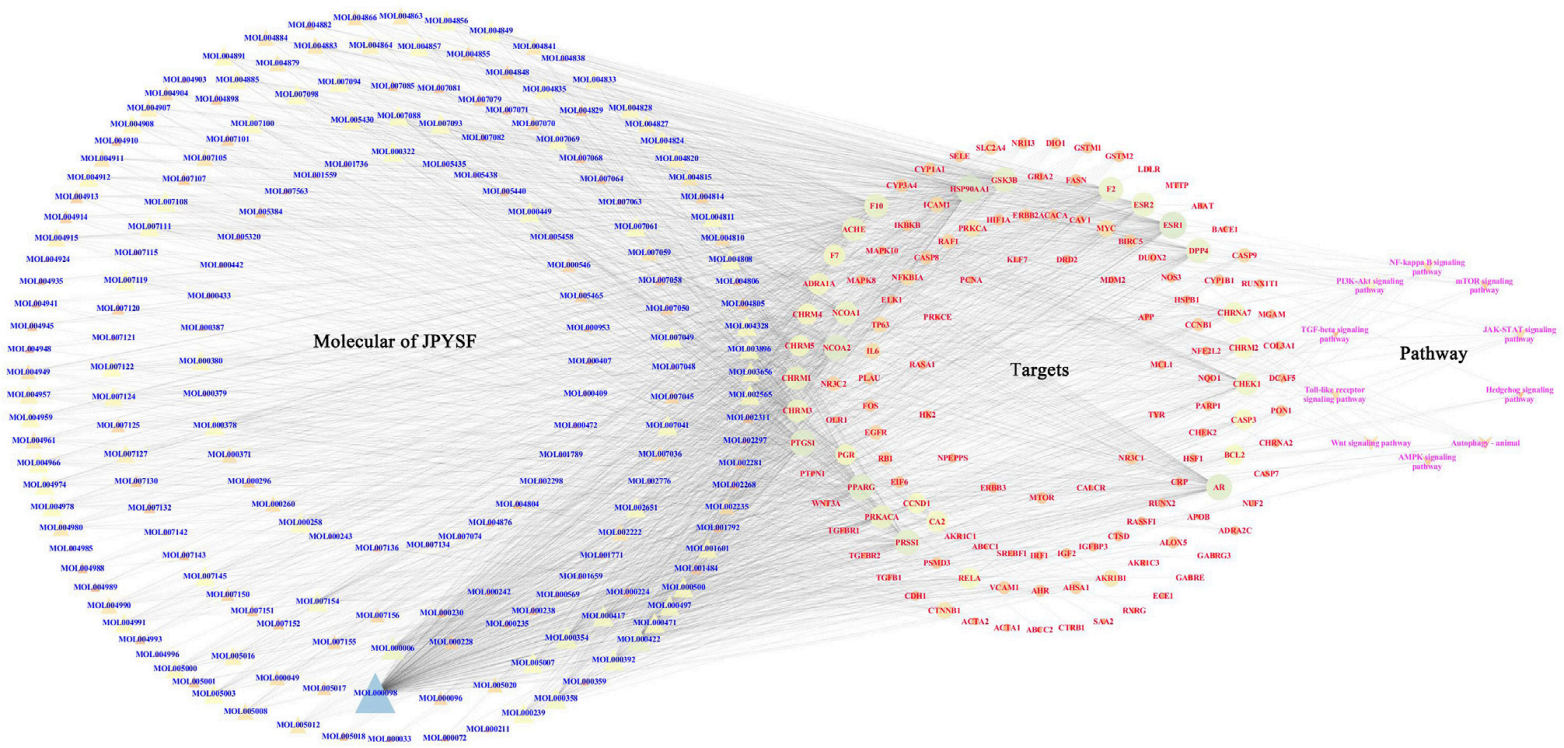
The “chemicals-shared target genes-signal pathway "(C-T-S) network. The blue fonts represent the components of JPYSF, the red fonts represent the targets of JPYSF components and epithelial–mesenchymal transition (EMT), and the rose fonts represent the signaling pathway.

**FIGURE 5:**
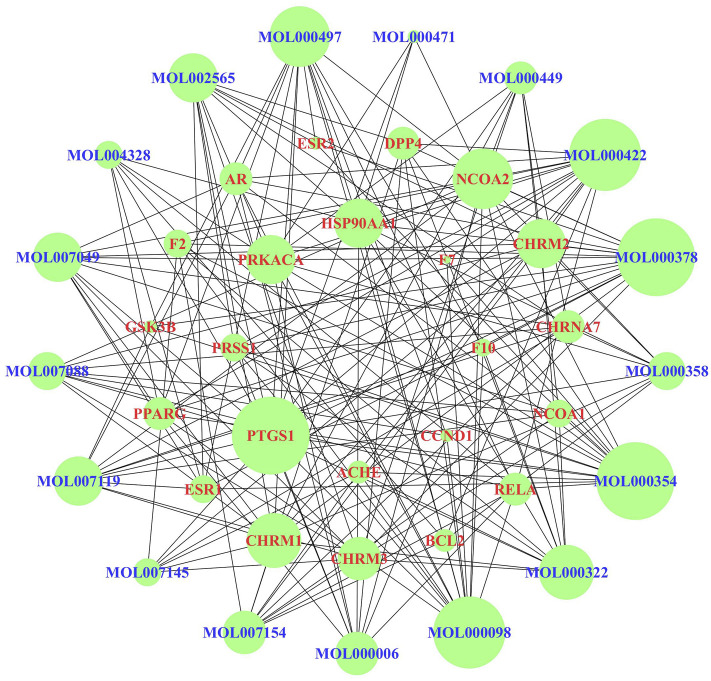
The core targets and compounds in the “chemicals-shared target genes-signal pathway” (C-T-S) network. The nodes with a larger area have a larger degree value.

According to the KEGG enrichment analysis results, the network of CTS was established to systematically explain the mechanism of JPYSF on EMT. There were 343 nodes that interacted with other nodes in the network. The rose font represented the top 10 pathways, the blue font represented the bioactive components, and the red font represented the common target genes of JPYSF and EMT (Figure 4). According to the topological parameters results of the network, the average degree value was 12.6006, the average betweenness centrality value was 0.0060, and the average closeness centrality value was 0.3365. In the network, the nodes with the topological parameters above the average degree, betweenness centrality, and closeness centrality of the C-T-S network were the core targets and the core chemicals of JPYSF in the treatment of EMT to relieve renal fibrosis (Figure 5). The core targets were NCOA2, HSP90AA1, PTGS1, PRKACA, ESR1, GSK3B, PPARG, AR, PRSS1, F2, F10, ACHE, CHRM1, DPP4, NCOA1, RELA, CHRM3, ESR2, CCND1, F7, CHRM2, BCL2, and CHRNA7. Core molecules included quercetin (MOL000098), kaempferol (MOL000422), luteolin (MOL000006), naringenin (MOL004328), isorhamnetin (MOL000354), Tanshinone IIA(MOL007154), aloe-emodin (MOL000471), stigmasterol (MOL000449), beta-sitosterol (MOL000358), salviolone (MOL007145), cryptotanshinone (MOL007088), licochalcone A(MOL000497), 7-O-methylisomucronulatol (MOL000378), kadsurenone (MOL000322), miltionone I(MOL007119), 4-methylenemiltirone (MOL007049), and medicarpin (MOL002565).

### Jian-Pi-Yi-Shen Formula Administration Significantly Reduced Epithelial–Mesenchymal Transition by Inhibiting the Wnt3a/β-Catenin Pathway in Renal Fibrosis of 5/6 Nx Rats

**FIGURE 6:**
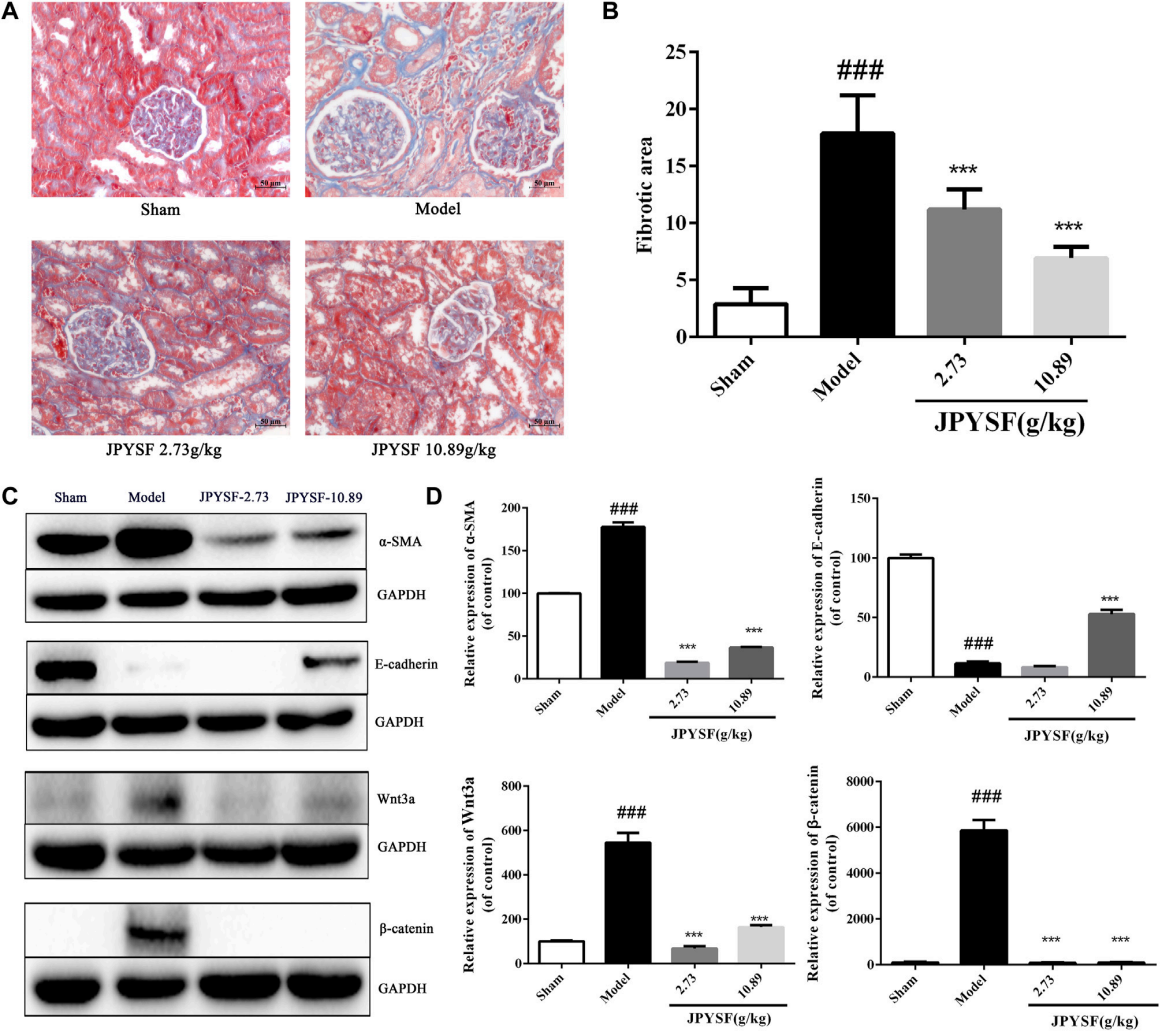
JPYSF administration significantly reduces renal fibrosis by alleviating EMT in 5/6 nephrectomy (Nx) rats. **(A)** Representative images of renal tissue are shown by Masson staining from the sham group, model group, the JPYSF 2.73 g/kg group, and the JPYSF 10.89 g/kg group (200×). **(B)** Quantitative analysis of the fibrotic area. Data were presented as the mean ± SEM, *n* = 10 rats per group (^###^, *p* < 0.001, compared with the sham group; ***,*p* < 0.001, compared with the model group). **(C)** Relative protein expression of α-SMA, E-cadherin, Wnt3a, and β-catenin in the kidney cortex tissues of the sham, model, 2.73, and 10.89 g/kg Jian-Pi-Yi-Shen formula (JPYSF) groups by western blot analysis. Representative bands show the relative expression of α-SMA, E-cadherin, Wnt3a, and β-catenin. **(D)** Quantitative analysis of protein expressions of α-SMA, E-cadherin, Wnt3a, and β-catenin by densitometry based on western blot images. ^###^,* p* < 0.001, compared with the Sham group; ***, *p* < 0.001, compared with the model group.

As shown in Figures 6A,B, 5/6 Nx rats showed obvious interstitial fibrosis by extensive blue staining of the tubulointerstitial area, which was 6 times that of the sham group in quantitative analysis (*p* < 0.001). Tubular atrophy and interstitial fibrosis in 5/6 Nx rats were significantly ameliorated in both 2.73 g/kg and 10.89 g/kg JPYSF groups.

To investigate whether JPYSF improved kidney fibrosis by restraining EMT, the protein expression of α-SMA and E-cadherin in the kidney tissues of 5/6 Nx rats was detected by western blot. As shown in Figures 6C,D, the results indicated that the expression of α-SMA was significantly up-regulated, and E-cadherin was reduced in 5/6 Nx rats compared with the sham group. The expression pattern was reversed by JPYSF administration in 5/6 Nx rats. In both 2.73 and 10.89 g/kg doses of JPYSF groups, the protein expression of α-SMA was decreased than that in the model group; while E-cadherin expression was increased than that in the model group by JPYSF administration at 10.89 g/kg. According to the analysis results of network pharmacology, the highest number of targets was in the Wnt signaling pathway and the most significant *p* value was in the enriched anti-fibrosis signaling pathway. Therefore, the protein expression of Wnt3a and β-catenin was calculated. The protein levels of both Wnt3a and β-catenin were increased in the model group than those in the sham group, and the changes were significantly reversed by the JPYSF treatment. The western blot results showed that JPYSF administration significantly alleviated EMT by inhibiting the Wnt3a/β-catenin pathway.

## Discussion

JPYSF, a classic prescription of hospital preparations, has been used clinically to treat renal fibrosis as an effective and safe TCM. Previous studies confirmed that JPYSF significantly decreased fibrotic area to ameliorate interstitial fibrosis and down-regulated fibrosis-associated protein expression such as fibronectin and type IV collagen in 5/6 Nx rats (Liu et al., 2018; Lu et al., 2018; Chen et al., 2019). These results indicated that JPYSF could inhibit EMT, one of the main mechanisms of renal fibrosis. However, the bioactive components and multi-target mechanisms of JPYSF on EMT have not yet been fully studied. It is therefore of great importance to comprehensively investigate the pharmacological mechanisms of JPYSF on EMT to alleviate renal fibrosis.

First, network pharmacology was employed to explore the potential active ingredients and targets of JPYSF. Second, the common targets were the intersection targets between the potential targets of JPYSF and EMT-associated targets, and then they were identified to build a protein interaction network. According to the string database, the top 30 targets with action frequency are shown in Figure 1C. Some genes, such as HSP90AA1 and RELA have been associated with the pathogenesis of EMT. Studies have found that HSP90AA1 exhibited anti-hepatic fibrosis effects in LX-2 cells (Chen CH. et al., 2021), and EMT was reported to be suppressed by RELA/p65 through integrin-mediated signaling (Roupakia et al., 2021). Third, the GO functional and KEGG enrichment analyses were carried out to further understand the mechanism of JPYSF. The enrichment analysis results predicted that the therapeutic effects of JPYSF against EMT might be involved in anti-fibrosis (the Wnt signaling pathway, the JAK-STAT signaling pathway, the Hedgehog signaling pathway, and the TGF-beta signaling pathway), anti-inflammation (the PI3K-Akt signaling pathway, the NF-kappa B signaling pathway, and the Toll-like receptor signaling pathway), podocyte protection (the Autophagy–animal and the mTOR signaling pathway), and metabolism regulation (the AMPK signaling pathway). In accordance with previous studies, these results indicated that the above pathways had important effects on the progression and advancement of EMT. Among them, the Wnt/β-catenin pathway is the best researched anti-fibrosis pathway related to EMT (ChenQ et al., 2021; Gao, et al., 2020). Therefore, the Wnt/β-catenin pathway may play a crucial role in JPYSF against EMT. Other anti-fibrosis signal pathways were also reported in the EMT process. For instance, EMT and glomerulosclerosis in rats with chronic kidney disease were suppressed by JAK/STAT signaling pathway (Zhao et al., 2019), and cyclosporine-A-induced renal fibrosis was ameliorated by chrysin by the inhibition of TGF-beta-induced EMT (Nagavally et al., 2021). In addition, the PI3K-Akt signaling pathway, a major regulator of anti-inflammation, has been reported to prevent renal fibrosis by attenuating renal tubular epithelial cell–mesenchymal transition (Hu et al., 2021; Zhang Q et al., 2020; Wang et al., 2019). In line with this, JPYSF inhibited the inflammation via suppression of the NF–kappa B signaling pathway (Lu et al., 2018). Then, the network of CTS and core CTS were performed to comprehensively predict the core compounds, target genes, and mechanism of JPYSF on EMT, as shown in Figures 4, 5. Degree centrality is the most direct and important parameter of the node among the three topological characteristic parameters. The node with a greater degree is more important in the network. The closeness between a node and other nodes in the network was reflected by closeness centrality. The node that is closer to other nodes has greater proximity centrality. Between centrality means the number of shortest paths through a node. The results of the topological analysis of the C-T-S network showed that 17 core components and 23 core targets had higher values than the average degree centrality value, closeness centrality value, and between centrality value. All the compounds that might participate in the regulatory processes of JPYSF in EMT were considered as potential active ingredients. In support of this, several previous studies have shown that quercetin (Elumalai et al., 2021), kaempferol (Ji et al., 2020), luteolin (Cao et al., 2020), naringenin (Li et al., 2021), isorhamnetin, Tanshinone IIA (Fu et al., 2021), cryptotanshinone (Zhang Z et al., 2020), and aloe-emodin (Liu et al., 2021) had well-established roles in EMT*.* Taken together, our results demonstrate that the core components may be the effective ingredients of JPYSF for the EMT treatment.

Finally, the 5/6 Nx-induced renal fibrosis rat model was selected to evaluate the therapeutic effects of JPYSF on EMT to verify the network pharmacology prediction. As expected, JPYSF treatment significantly ameliorated interstitial fibrosis in 5/6 Nx rats by Masson staining analysis (Figure 6). In our previous studies, we have reported that JPYSF notably reduced the expression levels of fibronectin and type IV collagen by immunofluorescence analysis in renal fibrosis of 5/6 Nx rats (Liu et al., 2018). We, therefore, expect a similar outcome that the other markers of fibrosis can be identified. Furthermore, α-SMA, a mesenchymal cytoskeletal marker, and E-cadherin, an intercellular epithelial adhesion molecule, were selected to assess whether JPYFS had a modulating effect on EMT. The Wnt/β-catenin pathway, the best-enriched anti-fibrosis pathway related to EMT, was chosen to test the predicted molecular mechanisms and verify the accuracy of network pharmacology prediction. Consistently, the western blot analysis detected that JPYSF markedly up-regulated expressions of E-cadherin, Wnt3a, and β-catenin, and down-regulated expression of α-SMA in 5/6 Nx rats. In agreement with this, our previous studies have reported that JPYSF notably reduced the increased levels of fibronectin and type IV collagen in renal fibrosis of 5/6 Nx rats (Liu et al., 2018). Moreover, it has been reported that astragaloside IV up-regulates the decreased expressions of E-cadherin and occludin, whereas it down-regulates expressions of N-cadherin and vimentin in high glucose-induced EMT cells (Wang YN et al., 2020). Therefore, we assume that JPYSF could regulate other epithelial markers and mesenchymal markers. To support this notion, astragaloside IV, which is an active ingredient from *A. mongholicus*, is considered as the monarch drug, the ingredient that provides the principal curative action on the main syndrome in JPYS prescription and has also been identified within JPYSF by HPLC-MS analysis in the current study (Supplementary Figure S1). Since most of the EMT markers are cell- and drug-treatment-specific, checking the expression of EMT markers in the specific context by JPYSF would be the future potential work. Collectively, these results provide evidence that JPYSF could regulate EMT by inhibiting the Wnt3a/β-catenin pathway.

However, there were some limitations of research, such as the core compound with no effective dose, which still needed further experiments to identify. Moreover, the direct regulatory relationship of JPYSF in EMT and the Wnt3a/β-catenin pathway by gene knockout animal experiments and cell experiments would be our future potential work for a better understanding of this signaling pathway.

## Conclusions

**FIGURE 7:**
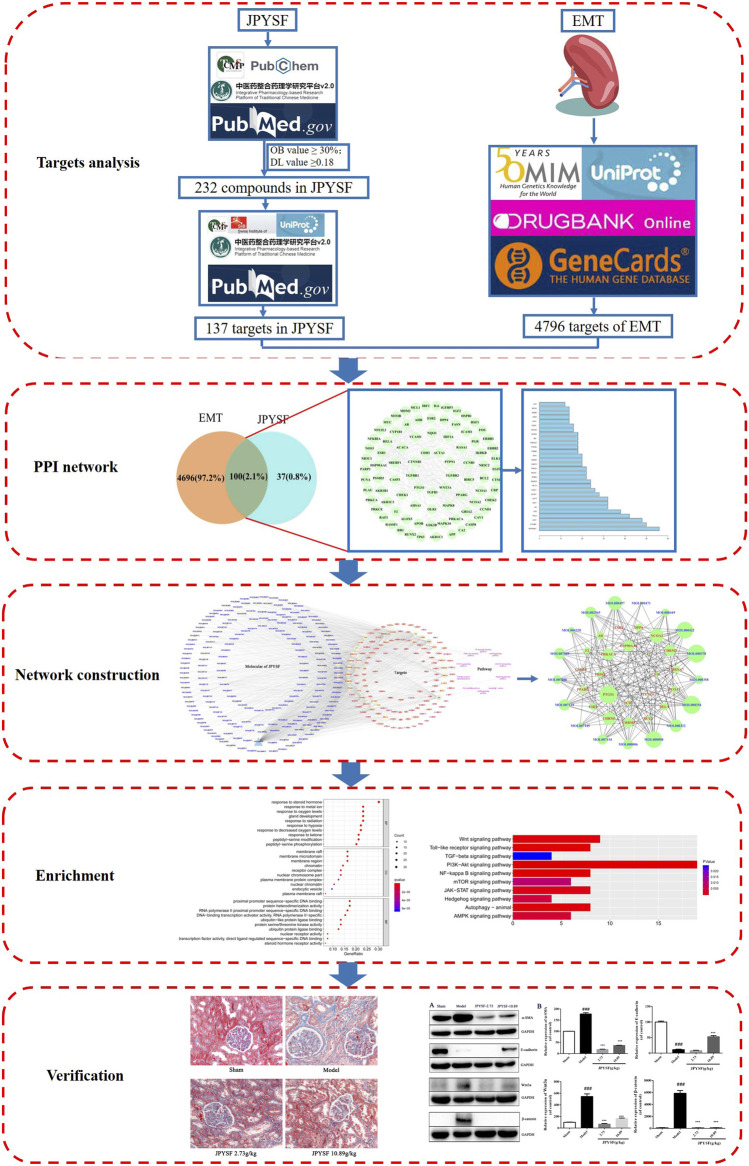
The workflow of the system’s pharmacology analysis and experimental verification of Jian-Pi-Yi-Shen formula (JPYSF) in suppressing epithelial–mesenchymal transition (EMT).

In this research, a combining approach of systematic network pharmacology and experimental verification were employed to determine therapeutic targets and pharmacological mechanisms of a complex herbal formulation, JPYSF, against EMT. The results suggested that 17 core compounds such as quercetin, kaempferol, luteolin, naringenin, and 23 core targets such as NCOA2, HSP90AA1, and PTGS1 might play an important role in JPYSF-treated renal fibrosis. The multi-target synergetic mechanisms of JPYSF in EMT mainly consist of four therapeutic aspects including anti-fibrosis, anti-inflammation, podocyte protection, and metabolism regulation. *In vivo* experiment data showed that JPYSF significantly ameliorated interstitial fibrosis by Masson staining and exerted the regulation of EMT by inhibiting the Wnt3a/β-catenin pathway (Figure 6). In summary, our study provides a basis and guidance for JPYSF research and its clinical application in the treatment of EMT, offering insights into the multi-target mechanisms by the system’s pharmacology approach (Figure 7).

## Data Availability

The original contributions presented in the study are included in the article/Supplementary Material, further inquiries can be directed to the corresponding author.

## Supplementary Material

Supplementary Figure S1 HPLC-MS chromatograms of JPYSF extract. The notations are as follows: 1: Acteoside; 2: Calycosin 7-O-glucoside; 3: Liquiritin; 4: Rosmarinic acid; 5: Salvianolic acid A; 6: Calycosin; 7: Astragaloside IV; 8: Formononetin; 9: Rhein; 10: Dioscin; 11: Atractylenolide I; 12: Tanshinone IIA.

## Abbreviations

JPYSF, Jian-Pi-Yi-Shen Formula; EMT, epithelial–mesenchymal transition; 5/6 Nx, 5/6 nephrectomy; TCM, traditional Chinese medicine; CTS, chemicals-shared target genes-signal pathway; BP, biological process; CC, cell component; MF, molecular function; SD, Sprague–Dawley.
