# Supplementary figures and images for: Network Pharmacology and Experimental Verification Strategies to Illustrate the Mechanism of Jian-Pi-Yi-Shen Formula in Suppressing Epithelial–Mesenchymal Transition
Source: Front Pharmacol. 2022 May 17;13:873023. doi: 10.3389/fphar.2022.873023 (PMC9152215; doi:10.3389/fphar.2022.873023)

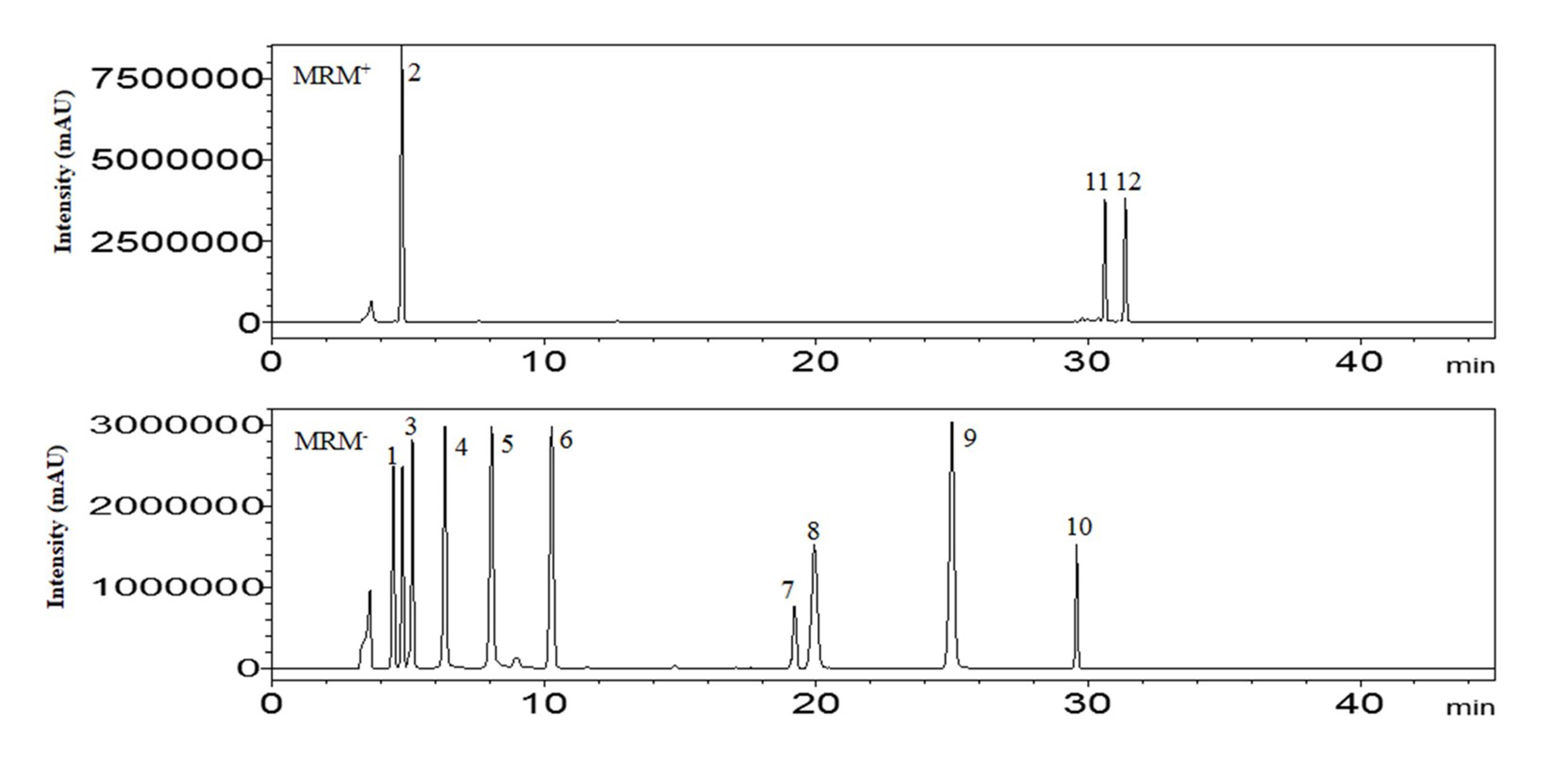

Supplement: Supplementary file 1 [file Image1.JPEG]
